# Children’s perceptions of factors influencing their physical activity: a focus group study on primary school children

**DOI:** 10.1080/17482631.2021.1980279

**Published:** 2021-10-18

**Authors:** Gabrielle Wann Nii Tay, Mei Jun Chan, Gayatri Kembhavi, Jubilee Lim, Salome A. Rebello, Hazyl Ng, Congren Lin, Lynette P. Shek, Carla Lança, Falk Müller-Riemenschneider, Mary Foong-Fong Chong

**Affiliations:** a Saw Swee Hock School of Public Health, National University of Singapore, Singapore; b Centre for Evidence and Implementation, Singapore; c Health Promotion Board, Singapore; d Department of Paediatrics, Yong Loo Lin School of Medicine, National University of Singapore, Singapore; e Singapore Institute for Clinical Sciences, Agency for Science, Technology and Research, Singapore; f Singapore Eye Research Institute, Singapore

**Keywords:** Perspectives of children, physical activity, motivations, qualitative research, socio-ecological model

## Abstract

**Purpose:**

Establishing healthy lifestyle behaviours in primary school children is important, as these behaviours are likely to track into adulthood. This study aimed to explore the factors influencing physical activity (PA) in primary school children through their perspectives.

**Approach:**

Eleven focus group discussions and one interview were conducted with 52 children (n = 29 girls) aged 9–12 years from two primary schools in Singapore. Data analyses were conducted using thematic analysis, deductively following the socio-ecological model (SEM) and inductively for themes at each SEM level.

**Results:**

At individual level, children’s perceived enjoyment, health benefits and expectation of rewards motivated them to engage in PA, while time constraints and their apathy towards PA hindered PA engagement. Children’s PA occasions at home were reported to be influenced by parental permission, priorities and availability, and the availability of preferred peers. Physical environmental factors such as opportunities for PA in school, access to facilities for PA and weather influenced children’s time spent on PA and the types of activities they engaged in.

**Conclusion:**

This study summarized some factors that children have reported to influence their PA behaviour. These findings could help inform future interventions aimed at promoting PA among primary school children in Singapore.

## Introduction

Physical activity (PA) is fundamental to optimal growth and development in children and adolescents (World Health Organization, 2020). Regular PA during childhood has shown to be associated with numerous physical and mental benefits for children, such as improved cardiorespiratory fitness and a reduced risk of experiencing depression and other mental health conditions (Chaput et al., 2020; Janssen & Leblanc, 2010). PA also helps prevent several chronic diseases, such as diabetes, cardiovascular diseases, and some types of cancers in the long term (Lee et al., 2012; Wu et al., 2019). While it is recommended that young people aged 5–17 years participate in at least 60 minutes of moderate-to-vigorous physical activity (MVPA) every day for its associated health benefits to be realized, current evidence demonstrates that more than two-thirds of young people do not engage in sufficient PA to benefit their health (Chaput et al., 2020; Guthold et al., 2020; Hallal et al., 2012). In Singapore, previous studies showed that children and adolescents only met up to 40% of the recommended PA levels (Chia, 2008), while in a recent study involving 233 adolescents, none of the adolescents achieved the recommended 60 minutes of MVPA (Ting et al., 2015). The low levels of PA among children, globally and locally, is a pressing issue, as PA levels tend to decline as children transit into adolescence (Nader et al., 2008; Ortega et al., 2013).

Evidence from previous studies suggests that this transition period is a critical period to intervene in children’s PA behaviours, as children begin to take responsibility for their PA participation (Wang et al., 2020). Additionally, health behaviours adopted at this stage have shown to track into adulthood (Craigie et al., 2011). For interventions to be effective, a better understanding of the factors influencing children’s PA behaviours is important. However, studies conducted to understand the determinants of PA behaviours among children often collect data from parents instead of children themselves. A growing body of literature has demonstrated discrepancies between parent reports and child self-reports of different aspects of the children’s lives—from their PA levels to their food intake, and even their emotional and behavioural problems (Thorn et al., 2013; Vereecken & Maes, 2014; Y.-Y. Chen et al., 2017). Because parental reports are less accurate, it is of utmost importance that information about children is obtained from the children themselves. Additionally, the active involvement of children in research projects allowed pertinent contextual input to be gathered (Jacquez et al., 2013). Consequently, this increased the chances of research findings being applicable to children in the real-world setting and be more effective when incorporated into intervention policies and programmes as demonstrated in the Girls Study Girls, Inc. (P. Chen et al., 2010) and OPT4College project (Rosen-Reynoso et al., 2010). The results of this study could be useful for future research aimed at designing and developing PA interventions and policies that would be appropriate for Singaporean children.

In recent years, there has been an increasing number of qualitative studies conducted to elicit the perspectives of children pertaining to their PA behaviours and influencing factors. These have mainly been conducted among children from Western populations such as New Zealand, England or the USA (Brockman et al., 2011; Carlin et al., 2015; Kirby et al., 2013; Moore et al., 2010; Pawlowski et al., 2018, 2014; Van Den Berg et al., 2018), with some studies conducted in the Asian context (Aoyagi et al., 2020; Wang et al., 2020). However, existing Asian studies are mostly from Chinese territories and do not report data from children of different ages and genders independently (Wang et al., 2020). Another conduced in Japanese adolescents focus specifically on school-based extracurricular sports participation (Aoyagi et al., 2020). We wanted to investigate if there are similarities or differences in perspectives of children from Singapore, a racially and culturally diverse country (Caprio et al., 2008; Long et al., 2012), and those from the existing Asian studies in relation to their PA behaviours both at home and in school. Moreover, we chose to focus on younger adolescents (aged 9 to 12 years), whose perspectives may differ from older adolescents, as they are less likely to have independent control of their PA choices (Doggui et al., 2021; Kneeshaw-Price et al., 2013). Additionally, by stratifying the focus groups by gender, we sought to examine if perspectives differing between gender exists.

A better understanding of the contextual and environmental factors influencing children’s behaviours, from children’s perspective, is pivotal in the design and development of effective, age and culturally appropriate lifestyle interventions and policies aimed at promoting PA in children in Singapore.

## Methods

### Participants

Focus group discussions (FGDs) and a semi-structured interview were conducted with primary school children aged between 9 and 12 years. Children in this age group were selected, as most children aged 7 years and above are capable of providing accurate and useful information when research methods that complement their developing competencies are employed (e.g., individual and group semi-structured interviews) (Christensen & James, 2008; J. E. Gibson, 2012). Moreover, research has shown that children, even very young children, enjoy being asked their opinions and are capable of adequately articulating their perceptions and beliefs (Christensen & James, 2008). In this study, children were recruited by convenience sampling from two public, co-educational primary schools and stratified by academic level into three groups (Primary 3 together with Primary 4, Primary 5, and Primary 6), gender and school. Parents provided written informed consent for their children to take part in the study, while the children provided written informed assent. Children who gave assent and whose parents gave consent to participate also provided demographic data when they submit their assent and consent forms. A total of 52 children, aged 9–12 years (mean ± SD: 10.8 ± 0.89) participated in the study. Slightly more than half (*n* = 29) of them were girls. Children who participated were of different ethnicities—Chinese (*n* = 39), Indian (*n* = 8), and Malay (*n* = 5). All the children participated in at least one co-curricular activity (CCA), which is not part of the standard school curriculum but typically conducted after school hours by the school and held in the school. Half of the children were involved in physical sports CCA (*n* = 26), the remaining were involved in performing arts (*n* = 12), clubs and societies (*n* = 7), and uniform groups (*n* = 6). One child was a member of both a performing arts CCA and a uniform group CCA.

### Data collection

A semi-structured discussion script was developed to aid the FGD and interview (Table 1). It was principally guided by the socio-ecological model (SEM), which describes the interactive characteristics of both individuals and environments, which form the basis of health outcomes (Golden & Earp, 2012). The script was designed to elicit information on the perceived factors influencing PA in primary school children at four different levels—individual, social environment, physical environment and macrosystem. Several activities, such as sorting picture cards, scenario-based questions (e.g., “What do you think will happen if a child is not physically active every day?”) and activity mapping (e.g., locations where children would usually go after school), were also incorporated into each FGD (see Supplementary Material). These techniques were used to sustain the children’s interest and concentration, help elicit their individual experiences, and provide them with opportunities to work collaboratively (J. E. Gibson, 2012; F. Gibson, 2007). A pilot-testing phase involving five FGDs with two different groups of children from the same schools were implemented about a month before data collection began. The purpose of the pilot testing was to assess the feasibility of activities planned, group characteristics (i.e., whether the focus groups should be mixed-gender or single-gender), and to determine if sufficient time was allocated for each activity. Based on the experiences and feedback from the pilot phase, adjustments were made to simplify the activities and the grouping of children (e.g., having single-gender groups, and seating children who were already friends apart to prevent them from engaging in conversation among themselves during the focus groups). Throughout the data collection process, field notes and memos were analysed by MJC, GWNT and JL after each FGD for the refinement of the phrasing of questions in the script (Golden & Earp, 2012; Pope et al., 2000).

**Table 1:** Key questions in the script for focus group discussions

SEM level	Key Questions
Individual	• What are your top 2 favourite activities and 2 least favourite activities? Why? *(Choose from pile of activity cards)*
	• What do you think about physical activity and physical education lessons? Do you think they are important?
Social environment	• Who do you usually play or exercise with?
	• Who are the people who would normally encourage/ discourage you to go out and play? Why do you think they would say this?
Physical environment	• Where do you usually go to play? Is the place near or far from your home?
	• Where else do you do other activities (e.g., home, playground)?
	• Why? Do you think it is important to spend some time outdoor?

A total of 11 different groups of children participated in the FGDs. Ten of these groups took part in two different FGDs, with one focusing on factors influencing their PA, and the other focusing on factors influencing their dietary choices. Due to time limitations, one group could only participate in one FGD, where both PA and diet were discussed. Each focus group consisted of between 2 to 7 children, with a median number of 4. Although we aimed to have 4 to 6 children in each group, some focus groups had fewer children due to low response rates and absentees. As a result, a semi-structured individual interview instead of FGD was conducted with one child. This work will report on findings of the FGDs about influences on PA. For this study, we decided to focus on PA in relation to sports, exercise, and active play, but not include transportation or sedentary behaviour.

The FGDs and one interview took place after school hours within the school compound in a room with minimal noise and distractions (e.g., a room in the school library). For each FGD, the children sat in a circular arrangement, which allowed for the two moderators to sit among them and create a non-authoritarian climate (F. Gibson, 2007). Two researchers were present at each FGD and the interview; other adults such as teachers and parents were not involved. All sessions were conducted in English, which is the primary medium of instruction in all schools in Singapore. All FGDs were conducted by MJC who received training and guidance on the conduct of FGDs with children from an experienced qualitative researcher (GK). An assistant moderator (GWNT or JL) attended the FGDs to help with notetaking and time management. There were no pre-existing relationships between all researchers and the children. Ground rules for the FGDs were discussed (e.g., respect the opinions of each child and each child should “say what [they] think”, rather than what they thought others wanted to hear). The children were also informed that there were no “right or wrong opinions” and their responses would be kept confidential. An icebreaker question (“Tell me one interesting fact about yourself”) was asked before starting each FGD. Each FGD was recorded using Sony UX560F digital voice recorders and lasted approximately 60–100 min (median: 78 min). All participants were presented with tokens of appreciation (e.g., stationery and snacks) for their time. Ethical approval for all data collection procedures was obtained from the University’s institutional review board [reference number: S-18-087]. Approval was also obtained from the Ministry of Education to approach schools for recruitment [reference number: RQ17-18(02)].

### Data analysis

All recordings were transcribed verbatim without identifiers and imported to NVivo (Version 12, QSR International) to assist the organization of data. Two researchers (MJC, who was trained in qualitative research analysis, and GWNT) analysed and coded the transcripts using thematic analysis (Braun & Clarke, 2006), focusing on the contextual factors influencing children’s PA behaviours. Both researchers read all the transcripts repeatedly, coded the data deductively based on the SEM and then inductively for sub-themes in each level of the SEM. Coding of data was done independently, and researchers met at regular intervals to discuss issues with codes and emergent themes until consensus was reached. Final themes were generated after further discussions and deliberations between both researchers and MFFC to ensure that different perspectives were covered (Smith & Mcgannon, 2018).

## Results

**Figure 1.**
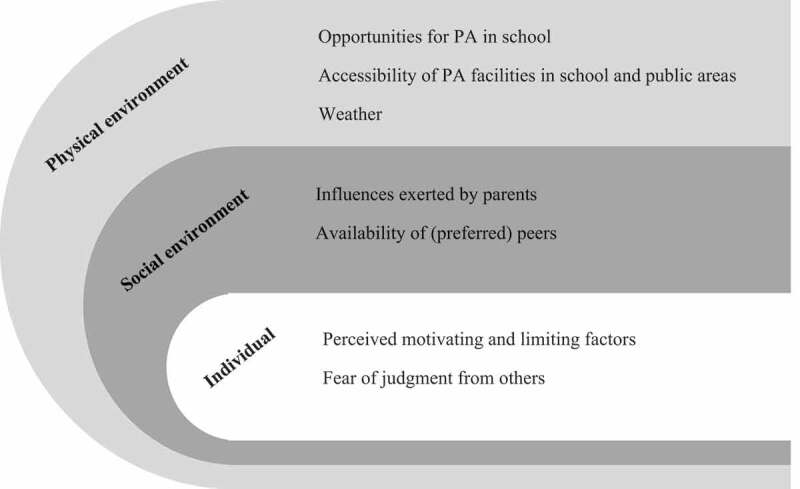
Summary of themes categorized into different levels of the SEM

Key themes and subthemes that emerged from the data pertaining to the influences of PA in primary school children have been classified according to the three levels of the SEM—the individual, social environment, and physical environment, as shown in Figure 1. Overall, the analysis of the focus group data yielded themes that were comparatively similar between genders and across ethnicities, except for the theme “Weather”, where the opinions between girls and boys differed. Themes were also similar across ages, but responses from younger children tended to be more straightforward and literal.

### Individual influences

#### Perceived motivating and limiting factors

This theme includes the individual factors that both stimulated and restricted PA among children in our study.

##### Perceived enjoyment

Most children expressed that they preferred to engage in PA that they perceived to be enjoyable. Enjoyment was most often derived from having fun and in some cases, from them being able to relax, have their mood lifted or engage in activities they found entertaining. As one child said: “*I like dancing because I can exercise at the same time. I can entertain myself. Sometimes when I’m sad, dancing makes me happy*” [Girl, 12 years old]. Some also shared that they would not participate in certain forms of PA that they did not enjoy: “*I learned dancing from my friends, and I failed really badly. I was embarrassed in front of my friends, so I didn’t do it again*” [Boy, 11 years old], “*I used to play basketball in my school with my friends and I always got hit on the head* [Girl, 9–10 years old]”. This emphasizes the idea that the enjoyment derived from engaging in PA is a strong facilitator in primary school children. Conversely, being asked to participate in forms of PA they do not enjoy may be a limiting factor for PA.

##### Perceived health benefits and expectations of rewards

Many children shared that they understood the physiological and psychological benefits of PA, especially in relation to health in the longer term, yet few were actually motivated by this knowledge to engage in PA. The children also demonstrated knowledge of specific benefits of PA, such as improved physical appearance, weight control and opportunities to bond with friends. For example, “*I think it’s quite important ‘cause you cannot be studying (for) the whole day and not going out to exercise or play because you can’t get vitamins you need from the sun and … you need to get some exercise, not only to keep fit but to take your mind off the stress or studying, like the problems you have in school*” [Girl, 12 years old]. In contrast to this understanding, a few children reported that they were willing to engage in PA because of the food rewards that would follow, most often fast food or less healthy snacks. As mentioned by one child, “*If I want to go to a park, I will go to West Coast. Then after that, we will eat at McDonald’s*.” [Girl, 9–10 years old].

##### Perceived time constraints

Many children expressed that they struggled to allocate time for regular PA in their schedules due to the academic obligations, which required them to spend a significant amount of time on homework, revising for examinations and attending private supplementary tutoring, known locally as “tuition” (Tan, 2017). This greatly reduced the amount of leisure time they had, with one child [Boy, 12 years old] saying: *“I don’t leave house that much because I have to study”*.

##### Apathy towards physical activity

Children shared that they were largely disinterested in engaging in PA, citing reasons such as “laziness” and a general preference for staying at home. A number of them, however, expressed their preference for staying at home particularly to engage in screen-viewing activities, such as playing video games, watching online videos, and watching television: “*I don’t want to leave the house … I am lazy to go out, lazy to find friends to play with. Just stay at home, sleep, watch TV*” [Boy, 11 years old]. Some also expressed that this disinterest resulted from them being physically tired from other activities such as tuition or enrichment classes. As one child explained, “*My mum wants me to go for tuition classes … sometimes I want to sleep but I still need to go, I feel so tired*” [Boy, 9–10 years old].

#### Fear of judgement from others

The fear of judgement by others, most often their peers, appeared to be both a barrier and, perversely, a motivator of PA in primary school children. Some expressed their distaste and reluctance towards engaging in certain activities, such as dance, as they felt self-conscious about how they would appear in front of others. They were also concerned about how others would perceive them, particularly if they made mistakes or when they felt that they were not “good enough”. As one child described, “*I’m scared (sic) I will make myself look stupid and then people will make fun of me*” [Girl, 12 years old]. Other children expressed that one of their motivations to engage in PA is to avoid being labelled by others, e.g., “being fat”, specifically by their peers: “*If you don’t keep fit, everyone will call you fat*” [Girl, 11 years old]. This reflects how the fear of judgement from others, intertwined with feelings of self-consciousness, can influence the decisions of some children to engage in certain PA.

### Social environment influences

#### Influences exerted by parents

This theme includes the significance of the influences of parents, who often have the most authority and power in the lives of children in this age range.

##### Parental permission to engage in physical activity

Outside the school setting, the engagement in PA among primary school children was largely contingent upon them being granted permission to do so by their parents. Children reported that this was because their parents were concerned about their safety. Some of these parental concerns include encounters with strangers, as well as the children getting injured from performing certain PA: “*She doesn’t allow me because she said it’s dangerous, (there are) strangers*” [Girl, 11 years old], “*I always (get) injured, that’s why my mum ask me don’t play any sports games*” [Boy, 11 years old]. Other children also mentioned that their parents felt that they were too young to go out on their own, without providing any further explanation to the children as to why they felt so. Conversely, some children shared that they were allowed to go out of the house on their own as long as they adhered to boundaries of distance and time set by their parents. As described by one child, “*For me, there’s a rule—I got to be back at a certain time or like, if I want to go anywhere else, I have to tell (my parents) before I go to that place and also for how long I’ll be there for*” [Boy, 11 years old]. A few children were also permitted to go out and play whenever they wanted, thus facilitating opportunities for greater PA.

##### Parental priorities

Some children mentioned that their parents had the tendency to prioritize their academic progress and performance or other activities requiring their involvement, such as gatherings with extended family members, over PA. Consequently, they would often be discouraged from engaging in PA, even if they had the desire to do so. One child revealed that “*Nowadays, I don’t really go out that much because I will need to study first*”, another child added “*that’s what happened to me even though I just want to play soccer right outside. (My mom is) always saying ‘do your homework first and then do my homework and tuition homework’*” [Boys, 10 years old]. On the other hand, many children who reported having regular PA as part of their daily schedules highlighted the role their parents played in the cultivation of this healthy habit—by constantly encouraging them to participate in PA.

##### Parental availability

Several children mentioned that they would typically engage in some PA together with their parents. Their parents would sometimes help them to master certain sports skills, such as cycling: “*I do cycling with my mother. My mother teaches me to cycle on two wheels*” [Girl, 11 years old]. A handful of children expressed that the frequency with which they would engage in PA was depended upon the frequency with which their parents were available to bring them out to sports facilities: “*I stopped going for swimming class because my father had an injury and cannot send me there*” [Girl, 10 years old]. This highlights the importance of parental availability as a facilitator of PA in primary school children.

#### Availability of (preferred) peers

Most children shared that they would engage in PA with their schoolmates, whether in or out of school. Some also had playmates living in the vicinity of their homes, with whom they would meet to play at nearby sports facilities: “*I play ball games like twice or once a week with my friends at the playground. And sometimes we also go around and play catching and do some climbing onto some high places and jumping down*” [Boy, 11 years old]. The availability of schoolmates or playmates had a significant influence on their engagement in PA; for some children, this was the only determinant of whether they actually engage in PA apart from the time scheduled for PA in the school curriculum. In addition, some children also highlighted that they would be less motivated to participate during school physical education (PE) lessons if they were grouped with peers that they were not particularly comfortable with, reinforcing the importance for children being able to engage in PA with the preferred company: “*It’s not that I don’t want PE, it’s because the people that we are grouped with are not good*” [Girls, 11 years old].

### Physical environment influences

#### Opportunities for physical activity in school

This theme serves to shed light on the existing opportunities (or lack thereof) for PA in schools.

##### Insufficient time for PA in school curriculum

While regular PE lessons are part of every school’s curriculum, some children detailed occasions where time spent on actual PA was considerably reduced when PE lessons did not start promptly, or if their teachers decided to discipline them due to their misbehaviour: “*If you misbehave then (the teacher) will take up the PE lesson just to scold us*” [Girls, 10 years old]. Most children also mentioned that the Physical Health and Fitness component of PE lessons (referred to as “Health Education” by children), which required them to be seated while learning the theoretical knowledge of a healthy lifestyle, reduced the time for engaging in more physically active activities, which they reported would have preferred to do. As children from one FGD revealed, “*I like PE for like 90%, and I don’t like PE for 10% because of the health education … It is this (workbook) on taking care of your eyesight and your health … And the worst part is when you have PE for one hour and teacher spends it on health education*” [Girls, 10 years old].

##### Programmes involving physical activity

Some children also shared, however, that they had other opportunities to engage in PA in school, apart from their weekly scheduled PE lessons. This included swimming lessons, which were part of the PE curriculum, as well as other school-specific enrichment Programmes like cycling or dance enrichment classes. Some also shared that they valued other school Programmes that took place outside of curriculum time and school-wide initiatives involving PA, as these created greater opportunities for PA within the school setting.

#### Accessibility of PA facilities in school and public areas

This theme includes the children’s perceived importance of certain characteristics of facilities for PA. When asked about the places that children would go to when they wanted to engage in PA, many of them shared that they would typically engage in PA at facilities that were either near their schools or their homes. Several children shared that there was an ease of access to sports facilities such as basketball courts, soccer fields and indoor sports halls in their schools, which generally encouraged them to be more physically active in school: “*I go to the soccer court near the school. Actually, it is also quite near my house*” [Boy, 11 years old]. Outside the school context, one child mentioned that the closure of the swimming pool near her home resulted in her discontinuing her swimming lessons and subsequently, her habit of swimming regularly: “*Because the swimming pool (near my house) was torn down, so my father was trying to look for other swimming coaches, but after that he said, ‘Ah never mind’*” [Girl, 10 years old]. Children reported that it was rare for them to travel any significant distance to engage in PA, thus the proximity of facilities for PA to school and home is an important facilitator of PA in primary school-age children.

#### Weather

Some children preferred staying indoors rather than going outdoors to play or exercise due to the availability of fans and air-conditioning indoors. Their preference was to stay indoors on a hot day. This preference for staying indoors also resulted from convenient access to drinking water, as well as access to shower facilities, which would help to alleviate the physical discomfort resulting from being sweaty. As mentioned by a child, “*The weather in Singapore is very hot. I like the (indoor) air conditioner more*” [Girl, 11 years old], another child added, “*Indoor it’s really convenient, you want water, it’s just there. You want to go do anything, it’s available indoor*” [Boy, 11 years old]. There was also mention of parental restrictions over going outdoors to play at certain times of the day, to avoid the heat and UV effects from the sun, although this was only a common theme among the girls: “*My mother says in the afternoon, it’s too sunny then I will (be) dehydrated or too tanned*” [Girl, 12 years old].

## Discussion and recommendations

This study aims to contribute to the limited body of literature pertaining to the factors influencing PA in primary school children, between 9 and 12 years of age living in Singapore, from their own perspectives. Our findings highlighted several themes at the individual, social environmental and physical environment levels of the SEM.

Perceived enjoyment, which was most often derived from having fun, was a strong motivator for PA among the children. This corroborates with the findings of a systematic review of qualitative studies exploring the barriers and facilitators relating to children’s PA conducted by Brunton et al. (2003), where fun and enjoyment were compelling reasons for the children’s participation in sports and exercise (Brunton et al., 2003). This idea is further supported by recent qualitative studies, which found that one of the main facilitators of PA was the enjoyment of activities (Abdelghaffar et al., 2019; Harvey et al., 2018; Van Den Berg et al., 2018; Wang et al., 2020). It is possible for this finding to be explained by the self-determination theory, which differentiates between autonomous and controlled forms of motivation (Ryan & Deci, 2017). Interventions targeting autonomous forms of motivation have been successful in changing specific PA-related behaviours among children between 5 and 18 years of age (Van Sluijs & Kriemler, 2016). To ensure sustained enjoyment rather than just momentary fun, it would be important to guide and provide children opportunities in identifying forms of PA that they would enjoy, both in and out of school, and subsequently encouraging and supporting their participation in such activities. This may also help reduce the apathy some children have towards PA.

While most children were aware of the myriad of benefits of PA for both physiological and psychological health, this awareness did not translate into PA-related behaviours. This finding differs from previous studies, which considered the awareness of the health benefits of PA to be a facilitator of PA for children (Abdelghaffar et al., 2019; Brockman et al., 2011; Van Royen et al., 2015). Brunton et al. (2003), however, found that most of the facilitators of PA in primary school children were focused on the “here and now”. The implications of PA on later adult health were rarely a motivating factor for these children to engage in PA, if at all. A possible explanation for this could be that children, at this age, are not able to fully comprehend what these implications mean to their health (Doherty & Hughes, 2009; Steinberg et al., 2009). Nevertheless, raising awareness and improving existing knowledge of the health benefits of PA among children is still important and necessary, as this knowledge still has the potential to motivate PA engagement and encourage behavioural changes (Butt et al., 2011; Vanhelst et al., 2017). Educational materials to enhance children’s knowledge could perhaps focus on short-term health benefits which children may find more relevant to them.

A noteworthy finding of our study was that a handful of children reported being motivated to engage in PA because of the food rewards (fast food or less healthy snacks) that would follow. This corroborates with the findings of previous studies, which demonstrated that rewards were effective in increasing PA engagement between the ages of 7 to 12 years (Hardman et al., 2011). However, fast food and snacks are often less healthy, and the excessive consumption of such foods can lead to weight gain and increase obesity risk (Yayan & Celebioglu, 2018). Previous studies also showed that using food to reward a desirable behaviour in children could foster their desire for these foods, and undermine their perceived importance of the behaviour (Fedewa & Davis, 2015). Thus, it would be important to consider alternative yet equally or even more enticing ways to reward PA engagement in primary school children.

Consistent with existing literature, our study found that a lack of time hindered the children from engaging in PA outside of school (Abdelghaffar et al., 2019; Carlin et al., 2015; Van Den Berg et al., 2018; Van Royen et al., 2015). Most of them associated their perceived time constraints with academic obligations, which greatly reduced their amount of leisure time at home. This corroborates with the findings of Abdelghaffar et al. (2019), where the children cited homework and prioritizing their educational achievements as factors that greatly reduced their leisure time and consequently, their participation in PA. To circumvent this, the authors suggested equipping children with skills to better manage their time, both in and out of school (Abdelghaffar et al., 2019). However, poor time management may not be the only underlying reason for the perceived time constraints among children in our study. The strong emphasis on academic achievements among children in Singapore and its competitive culture has resulted in many parents’ prioritizing academics over PA (Ng, 2020). As such, children and parents could perceive PA engagement as a factor that would negatively impact a child’s academic performance, and thus limit the time spent on PA. To encourage more PA among children, future interventions could consider framing PA promoting messages around the theme of improving learning abilities and academic performance (Te Velde et al., 2018; Wu et al., 2017).

The availability of social support has consistently been found to be a factor influencing PA in children (Abdelghaffar et al., 2019; Carlin et al., 2015; Kirby et al., 2013; Mendonça et al., 2014; Pawlowski et al., 2018). Indeed, we found that parents had a significant influence over the children’s engagement in PA in terms of parental permission, priorities, and availability. Children who reported having regular PA as part of their daily schedules shared that their parents had a role to play in accompanying and/or encouraging them to do so, thus reflecting the pivotal role of parents in influencing their children’s PA (Carlin et al., 2015; Sallis et al., 2000; Van Der Horst et al., 2007). Children also cited parental concerns regarding safety and their age as reasons for not being granted permission to go out on their own to engage in PA. However, similar to other studies, the most children perceived these concerns to be reasonable for their own protection (Brockman et al., 2011; Kirby et al., 2013; Thomson & Philo, 2004). It is interesting that safety concerns were being brought up by children as parents in a previous local study alluded that they felt their neighbourhood was safe for children to play in (B. Chen et al., 2020a). Findings from our study suggest that parents’ perception of safety may not be limited to physical safety (e.g., road and traffic) but may include social safety (e.g., people) as well. This supports the previous findings by Kemperman and Timmermans (2014) that social cohesion of the neighbourhood was also a factor of consideration when parents assess the safety of an environment for their children. Thus, to assuage the safety concerns of parents, community initiatives that facilitate social cohesion could constitute a part of future intervention Programmes. For example, getting children and their parents acquainted with other families living in the same or surrounding neighbourhoods. This could potentially result in a higher likelihood of permission being granted to the children for them to go outdoors and play on a (more) regular basis. However, further research is warranted to better understand parents’ concerns and their perceptions of a safe environment for their children to play in.

The importance of peers in the lives of children in this age range is well known (Carlin et al., 2015). A handful of children highlighted the availability of their peers, both from school and those living in the vicinity of their homes, as being an important factor in determining their engagement in PA outside of school. Friends are pivotal in making PA fun (Carlin et al., 2015; Corder et al., 2015), and thus children are more likely to be active when they are with their peers and friends in comparison to when they are alone (Harvey et al., 2018). This also accords to our earlier findings, in which fun and enjoyment strongly compel children to engage in PA. However, the availability of children and their peers to meet up to play is largely dependent on parental permission due to parents’ concern for their safety. Children in our study also reported being selective about the peers with whom they would engage in PA, preferably those whom they perceive to be encouraging and supportive. This suggests that considering the element of social interaction in PA interventions is equally critical.

When at school, children reported that there were opportunities created for greater PA engagement through events and programmes that were conducted, in addition to their weekly scheduled PE lessons. This finding adds weight to the notion that schools are suitable and important epicentres for the promotion of higher levels of PA (Pawlowski et al., 2018). However, in line with previous studies, children expressed their desire for more time to engage in physically active activities during PE lessons. This finding broadly supports that of Van Den Berg et al. (2018), who found that priorities given to PA during PE lessons depended strongly on the teacher (Van Den Berg et al., 2018). With the potential for PE to contribute to improving the level of PA in primary school children (Abdelghaffar et al., 2019; Carlin et al., 2015; Meyer et al., 2013), it is crucial that the allocated PE time in the school curriculum is maximized and this would require equal effort from both the children and teachers. A note of caution is due here, as these findings reflect the perspectives of children who attend public schools in Singapore and not those from private schools, which may follow a different curriculum framework and have different demographics of students (Ministry of Education, 2021).

We also observed a relationship between the ease of access to sports facilities and the children’s level of engagement in PA in the school context. The children shared that the ease of access to certain sports facilities encouraged them to be more physically active. This corroborates with the findings from a previous study, which found the ease of access to facilities to be a common facilitator of PA (Kirby et al., 2013). More specifically, another study conducted by Eime et al. (2017) found that the better provision of sports facilities was generally associated with increased sport participation. A possible explanation for this observation could be that PA becomes “normalized” when others are constantly seen utilizing these facilities (Macdonald, 2019). Many studies have also shown that interventions that target physical environment factors, in addition to personal and social environment factors, are more likely to be effective (Heath et al., 2012), thus highlighting a potential area of focus for future intervention programs.

Finally, our finding on the influence of weather is unique to the local context. As Singapore is situated near the equator, we experience a typical tropical climate with abundant rainfall, and high and uniform temperatures (Meteorological Service Singapore, 2021). Due to the hot and humid climate, children reported that they preferred staying indoors over engaging in PA outdoors, presenting weather conditions as a barrier hindering PA engagement among children. Additionally, children, especially girls, who are ethnic Chinese, also cited having tanned skin resulting from outdoor PA participation as a barrier for them. This socio-cultural barrier was also identified in previous studies conducted on ethnic Chinese children, who perceived lighter skin tones to be associated with being more attractive (H.-Y. Chen et al., 2020b; Wang et al., 2020), and were thus reluctant to play outdoors. This highlights the link of how the perception of beauty could influence outdoor PA engagement, or the lack thereof, in children. To help children overcome these barriers, alternatives to conventional outdoor PA engagement like doing indoor workouts and household chores (Butola et al., 2020) could be introduced to children during Health Education lessons in school.

## Strengths and limitations

Our study has several strengths and weaknesses. One strength of our study is that it focused on exploring the factors influencing PA in primary school children living in Singapore through their own perspectives, which contributes to a limited but growing body of literature (Foo et al., 2013). The second strength of our study is that the analysis of field notes and memos took place in tandem with data collection, which informed and allowed for the discussion script to be refined iteratively throughout the data collection process. This allowed for us to gather more in-depth data on specific topics of interest that were not previously considered (i.e., prior to data collection).

One main limitation of the study is that our findings may have limited transferability to other populations of children—for example, to children from private schools in Singapore or those with different social or cultural backgrounds. The system and environment of each school are unique, thus children from different schools would have different perceptions of the availability of time and resources for PA engagement in the school setting. However, our study aimed to engage in an in-depth exploration of the factors influencing PA in primary school children living in Singapore to inform the design and development of future interventions rather than to develop consensus among a larger population (F. Gibson, 2007). Also, most of the participants were of Chinese descent, it is possible that our findings may not reflect the views of children of Malay and Indian descent. Information on the children’s PA levels was not captured as part of this study, thus it is possible that children who were generally more enthusiastic about PA and had higher PA levels would have volunteered to participate in this study in comparison to those who were less enthusiastic. Lastly, although care had been taken to encourage truthful responses from participants, it is important to bear in mind that the perceived power differential between the researcher and the children may have encouraged the children to say what they thought the researcher wanted to hear (Christensen & James, 2008). Additionally, as younger children (9 to 10 years old) provided responses that were often more straightforward and literal, direct questioning was used more frequently with them, which could unintentionally have resulted in the use of leading questions.

## Conclusion

Overall, our study provided a summary of some of the factors influencing PA in primary school children living in Singapore, covering individual, social and environmental influences. Despite its exploratory nature, it offers insight to inform how lifestyle interventions and policies aimed at increasing PA among primary school children aged between 9 and 12 years old can be designed and developed, especially in the local context. Further research involving more children of different ethnicities is necessary to allow for a deeper examination of the specific impact of the individual influencing factors on participation in PA, as well as to provide greater insight into some of the emergent themes. Further research involving other stakeholders like the parents and teachers themselves, as well as environmental observations, are also warranted to triangulate and confirm these findings.

## Supplementary Material

Supplemental Material

## Data Availability

The datasets generated and/or analysed during the current study are available from the corresponding author on reasonable request.
