# Supplementary material for: Children’s perceptions of factors influencing their physical activity: a focus group study on primary school children
Source: Int J Qual Stud Health Well-being. 2021 Oct 18;16(1):1980279. doi: 10.1080/17482631.2021.1980279 (PMC8525992; doi:10.1080/17482631.2021.1980279)

# Supplementary Material

## Details of activity mapping

Using a set of activity cards, children were asked to create a map of where and what they do in school, after school and on weekends, with school located at the centre of the map. Location of activities were placed on the map based on how near or far they were from the school. This is followed up with a discuss the who and why they engaged these activities.

Example of map:


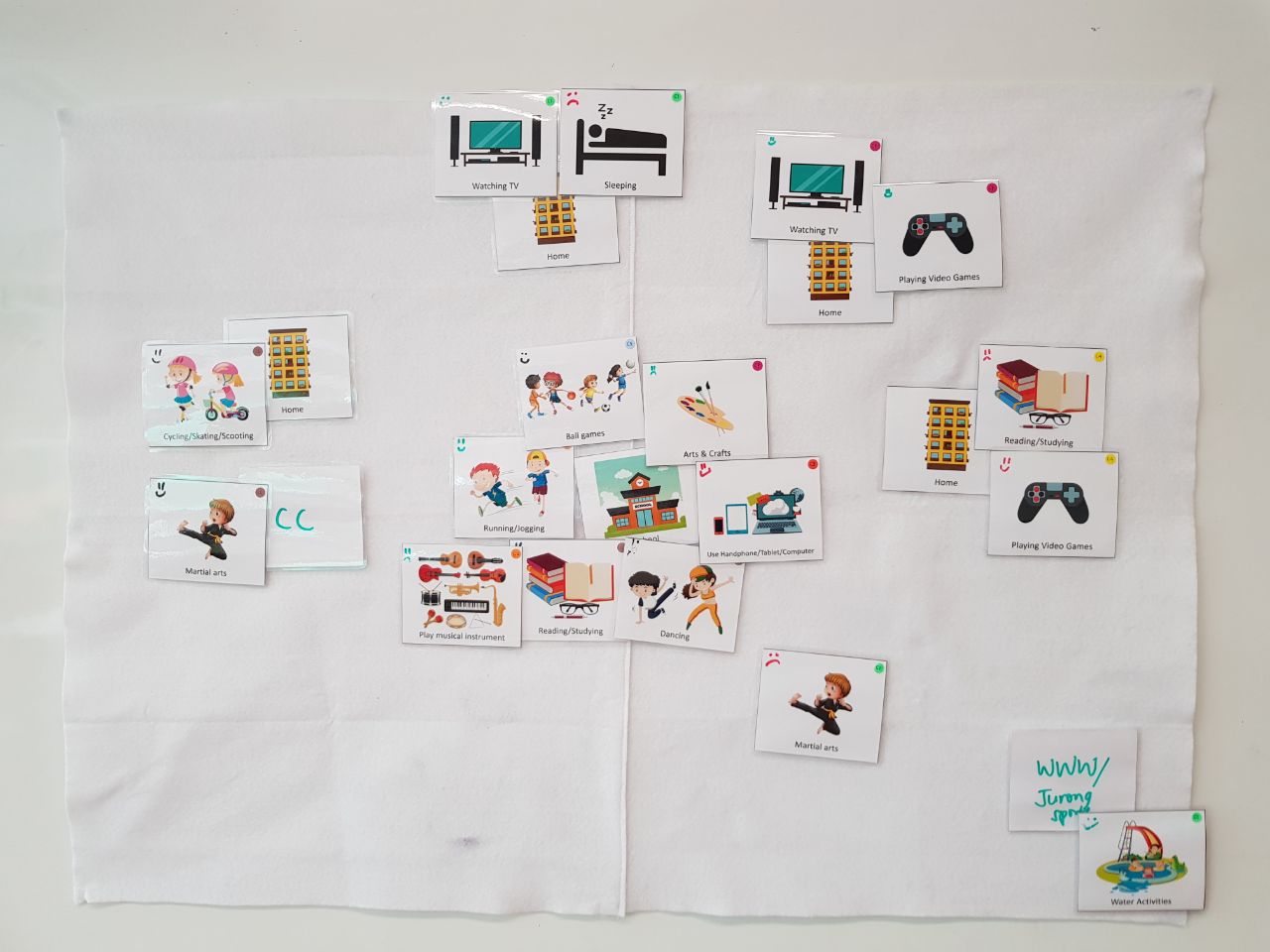


## Details of scenario-based questions

Children were presented with images of two scenarios illustrating children engaging in active and sedentary behaviour. They were then asked to discuss their activity preferences and their perceived importance of these behaviours.

Example of questions:

- Compare the 2 pictures here:
  - Which one would you rather do? Why?
  - Which one do you think is more important? Why?
  - What do you think will happen if you engage in activities in Image A every day? How about Image B?
  - Do you know how much physical activity child your age should do?


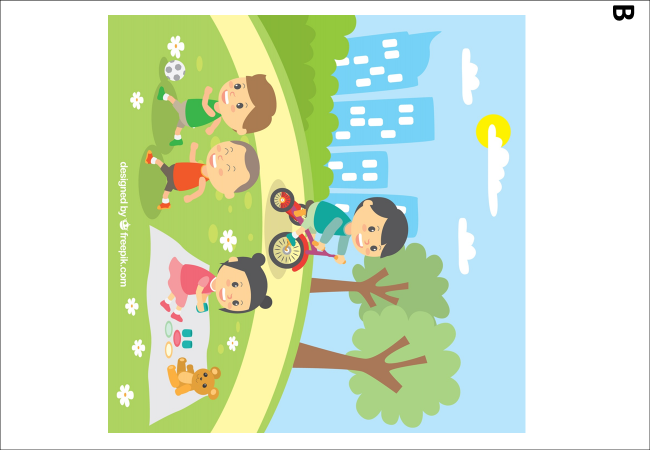

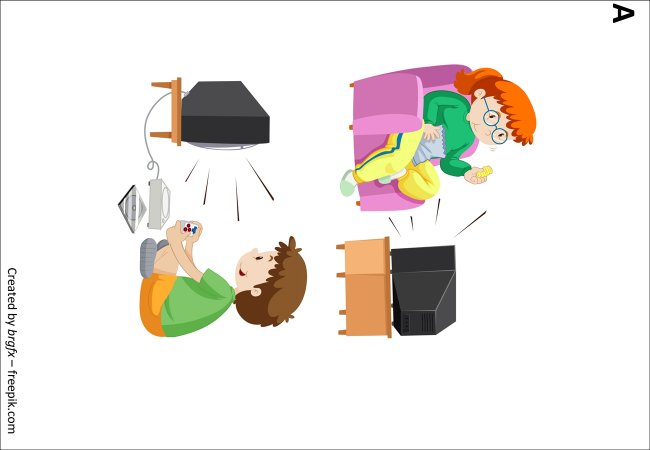

Supplement: Supplemental Material [file ZQHW_A_1980279_SM3579.docx]
